# Red Seaweed-Derived Compounds as a Potential New Approach for Acne Vulgaris Care

**DOI:** 10.3390/pharmaceutics13111930

**Published:** 2021-11-15

**Authors:** Adriana P. Januário, Rafael Félix, Carina Félix, João Reboleira, Patrícia Valentão, Marco F. L. Lemos

**Affiliations:** 1 MARE—Marine and Environmental Sciences Centre, ESTM, Instituto Politécnico de Leiria, 2520-641 Peniche, Portugal; rafael.felix@ipleiria.pt (R.F.); carina.r.felix@ipleiria.pt (C.F.); joao.reboleira@ipleiria.pt (J.R.); 2 REQUIMTE/LAQV, Laboratório de Farmacognosia, Faculdade de Farmácia, Universidade do Porto, 4050-313 Porto, Portugal; valentao@ff.up.pt

**Keywords:** acne pathophysiology, antibacterial, bioactive compounds, *Cutibacterium acnes*, inflammation, Rhodophyta, seaweed extracts

## Abstract

Acne vulgaris (AV) is a chronic skin disease of the pilosebaceous unit affecting both adolescents and adults. Its pathophysiology includes processes of inflammation, increased keratinization, sebum production, hormonal dysregulation, and bacterial *Cutibacterium acnes* proliferation. Common AV has been treated with antibiotics since the 1960s, but strain resistance has emerged and is of paramount concern. Macroalgae are known producers of substances with bioactive properties, including anti-viral, antibacterial, antioxidant, and anti-inflammatory properties, among several others. In particular, red algae are rich in bioactive compounds such as polysaccharides, phenolic compounds, lipids, sterols, alkaloids, and terpenoids, conferring them antioxidant, antimicrobial, and anti-inflammatory activities, among others. Thus, the exploration of compounds from marine resources can be an appealing approach to discover new treatment options against AV. The aim of this work is to provide an overview of the current knowledge of the potentialities of red macroalgae in the treatment of AV by reviewing the main therapeutic targets of this disease, and then the existence of compounds or extracts with bioactive properties against them.

## 1. Introduction

Acne vulgaris (AV) is a chronic disease of the pilosebaceous unit, and the most common skin disease in the world. It affects up to 85% of adolescents in the Occidental countries, and it can occur during adulthood in about 50% of people [1,2], having an incidence of nearly 9.4% of the worldwide population and being the eighth most prevalent disease in the world [3]. Acne deeply impacts quality of life through the manifestation of pimples, blackheads and scars on the face, upper chest and back, which can be painful and negatively affect the self-perception of beauty, leading to distress, anxiety, and depression [4,5,6]. Lesions associated with AV vary between non-inflammatory (white- and blackheads) and inflammatory (papules, pustules, and nodules), with different degrees of severity [5,6]. The main factors involved in acne pathogenesis are hormonal disequilibrium, exacerbated sebum production, follicular keratinization, and *Cutibacterium acnes* proliferation (formerly *Propionibacterium acnes*), resulting in an immune response and consequent inflammation [4]. Acne is the cause of 14% of primary care and 27% of dermatology consultations in the US, and first line treatment implies long periods of the use of antibiotics (up to three months, each time), oral and/or topical, usually complemented with retinoids, benzoyl peroxide, and oral contraceptives, among others [7]. In the last few years, *C. acnes’* resistance to the antibiotics most often used in acne treatment (erythromycin, clindamycin, and tetracycline, for example) has emerged [8,9]. Moreover, about 80% of treatments in women fail in multiple intake sequences of systemic antibiotics, and 30–40% fail after one course of the retinoid isotretinoin, one of the most successful drugs applied in cases of severe acne [10,11]. Therefore, the development of novel therapeutic approaches is of the utmost importance, for example, through the discovery of novel antibacterial and anti-inflammatory natural compounds.

Macroalgae are sessile marine organisms that live in a dynamic environment, being forced to adapt to fluctuations of biotic and abiotic factors, such as light, nutrients, salinity, temperature and predation. Seaweeds produce compounds to survive in response to those variations [12]. Moreover, marine macroalgae are considered to be among the finest sources of biologically active compounds of non-animal origin, having several valuable properties, including antioxidant, antimicrobial, anti-inflammatory, and antiviral properties [13], among others. Several classes of compounds are responsible for such bioactivities, e.g., proteins and peptides, poly- and oligosaccharides, fatty acids, sterols, phenolic compounds, pigments, vitamins, and minerals [14]. In particular, red algae species, the oldest division (Rhodophyta) of lower plants [15], are renowned producers of both anti-inflammatory metabolites (MAAs—mycosporine-like amino acids [16], phycocyanin [17], allophycocyanin [18], carotenoids [19], porphyrans [20]) and antimicrobials (diterpenes [21], monoterpenes [22], phenolic compounds [23], sterols [24], polysaccharides [25], fatty acids [26]). Because red seaweeds are so rich in bioactive compounds, including antibacterial and anti-inflammatory compounds, they seem to represent a promising source of natural, as opposed to conventional, anti-acne compounds or extracts. According to what has been mentioned, the goal of this review is to provide a compilation of compounds and extracts derived from red macroalgae that can hereafter be applied to AV research care.

## 2. Research Methodology

In order to compose this literature revision, Web of Science, Scopus, and Google Scholar databases were consulted up to 24 September 2021, using a Boolean search, and all of the publications identified from the following strings were analysed: (acne vulgaris AND (patho* OR inflamm* OR acnes OR epidermidis OR keratinization OR cornification OR comedo* OR hormone* OR sebum)) for Section 3; ((rhodophyta OR “red macroalg*” OR “red seaweed*”) AND (acnes OR epidermidis)) for Section 4.1; ((rhodophyta OR “red macroalg*” OR “red seaweed*”) AND (inflamm*)) for Section 4.2; and ((rhodophyta OR “red macroalg*” OR “red seaweed*”) AND (sebum OR hormon* OR testosterone OR androgen* OR estrogen* OR progesterone OR “growth factor*” OR “growth hormone*” OR hyperkeratinization OR cornification OR comedo*)) for Section 4.3.

## 3. Pathophysiological Targets for the Management of Acne Vulgaris

**Figure 1:**
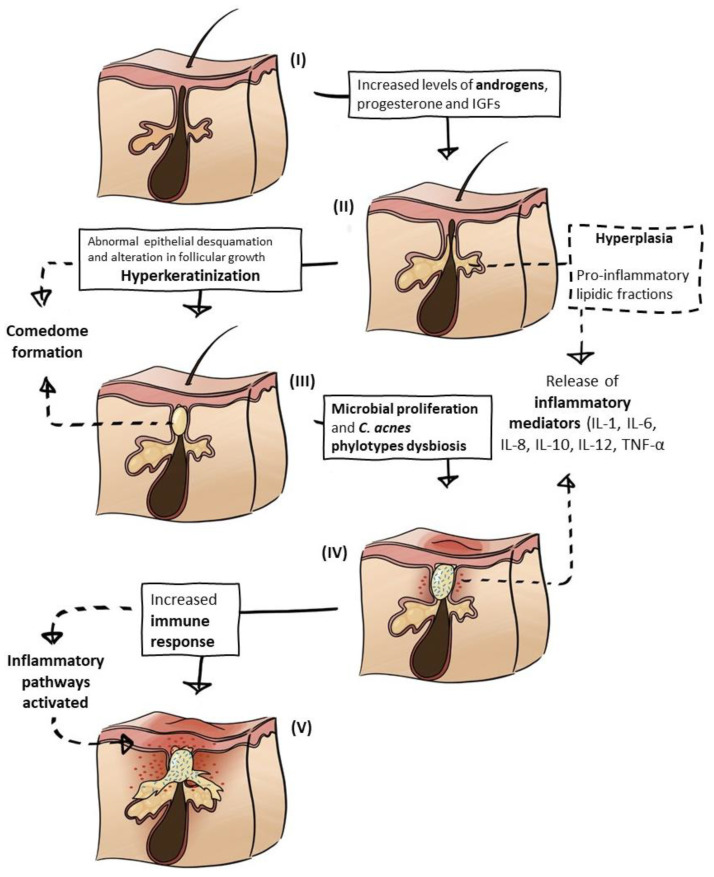
Acne vulgaris pathophysiology process schematics. (I) Pilosebaceous unit in normal conditions; (II) hormonal imbalance triggers seborrhea; (III) comedogenesis; (IV) microbial proliferation and inflammatory mediators’ release; (V) immune response followed by inflammation. The arrows represent the interplay between triggers.

A thorough literature analysis revealed that the general pathogenesis of AV is well established and amply described, but there is not yet a consensus regarding the whole pathophysiology, mechanisms, and order of events that lead to AV lesions. More recent reviews and research articles focus on the role of immune and inflammatory responses, bacterial dysbiosis, and the interplay between all of these factors [27,28,29], as opposed to older studies in which *Cutibacterium acnes*, sebum, and hormones were usually the main characters [3,30,31]. There have been several advances in the understanding of the role of each aspect in the cascade of events in AV, and the dominant factors and triggers that are interconnected in a highly complex manner are the following: (a) hormonal influence, mostly due to excessive androgen production and growth factors; (b) hyperseborrhea, triggered by the hormonal stimulation of the sebaceous glands; (c) comedogenesis, hyperkeratinization and the formation of comedones due to excessive sebum accumulation and abnormal epithelial desquamation; (d) microbial proliferation and dysbiosis, especially involving *C. acnes*, owing to a lipid-rich and anaerobic environment in the pilosebaceous unit; and (e) immune response and inflammation, as a consequence of pro-inflammatory molecules and activated pathways induced by the bacterium, hormonal disequilibrium, changes in sebum composition, and cytokine secreted by keratinocyte hyperproliferation and keratinization [3,4,6,28,29,30,32,33,34,35,36,37,38] (Figure 1). Acne-related factors are detailed below.

### 3.1. Hormonal Influence

Endogenous hormones—such as androgens, estrogen, progesterone, and insulin-like growth factors (IGFs), among others—play a critical role in the acne pathogenesis flow. Individuals that suffer from acne usually have high levels of androgens, progesterone, insulin, and IGFs, and, on the contrary, low levels of estrogen [6,39,40,41].

Androgens are the most important hormones controlling sebaceous gland activity, because they affect the sebaceous gland enlargement, sebocyte proliferation, and lipid metabolism, thus playing a crucial role in acne pathogenesis [42]. Free androgens are produced by the adrenal gland and gonads, but the most involved in acne are the androgens locally produced in the sebaceous glands. Inside the sebaceous glands is dehydroepiandrosterone sulphate (DHEAS), an adrenal precursor hormone that, by the action of steroid sulfatase, is converted into dehydroepiandrosterone (DHEA). The steroid metabolizing enzyme pathway expressed in the sebaceous glands seems to be initiated by 3β-hydroxysteroid dehydrogenase, which converts DHEA into androstenedione, then 17β-hydroxysteroid dehydrogenase converts androstenedione into testosterone, and finally, 5α-reductase converts testosterone into 5α-dihydrotestosterone (5α-DHT). DHT is three to ten times more potent than testosterone [43] when it comes to androgen receptors interaction, wherein the activity of 5α-reductase has a tremendous impact because its increased activity leads to the bigger size proportions of sebaceous glands. Furthermore, DHT increases lipidic production, not only by increasing the proliferation of sebocytes but also through the magnification of the mRNA levels of proteins involved in cholesterol, fatty acid, and triglyceride synthesis [6,44,45].

The growth hormone (GH) is secreted by the pituitary gland and can be found in sebaceous glands and hair follicles [46]. Diseases involving GH excess, such as acromegaly, are frequently associated with sebum overproduction, leading to acne development [44]. Furthermore, GH stimulates the production of IGFs, and, in particular, women evidencing acne lesions have higher levels of IGF-1, compared with androgens, sebaceous gland growth, and lipogenesis [42,47,48]. Naturally, IGF-1 levels tend to rise during puberty by the action of increased GH secretion, the same as for the levels of testosterone [49]. Features directly related to acne include the IGF-1 capacity to stimulate adrenal synthesis, resulting in increased androgen levels; IGF-1 also induces the proliferation of sebocytes; receptors of IGF-1 are expressed in sebaceous glands and hair follicles; IGF-1 mediates lipogenesis by the influence on the increasing expression of sterol regulatory element-binding proteins (SREBPs) transcription factor, which regulates the geneses involved in lipidic biosynthesis, and also by activating MAPK/ERK (mitogen-activated protein kinase/extracellular signal-regulated kinase) pathways; IGF-1 is able to stimulate 5α-reductase, sebocyte proliferation, and androgen receptor signal transduction [6,44].

For over six decades, oral contraceptive pills containing estrogens have been taken by women, not only as contraceptive method but also to suppress sebum production due to AV-related complaints [50]. Estradiol is the most abundant and potent estrogen. In particular, 17β-estradiol is produced through the aromatase enzyme into testosterone, and can be converted into estrone, a less potent estrogen, by 17β-hydroxysteroid dehydrogenase, with both enzymes being present in gonads, placenta, and skin [51]. Moreover, 17β-estradiol is the most potent sex steroid hormone, being 100 to 1000-times more potent compared to its androgen parents [52]. Estrogens’ roles include the influence on lower sebum segregation, including the opposition of androgens inside the sebaceous gland, because both hormones are able to activate the signaling pathways of estrogen receptor α (ERα), Erβ, and the androgen receptor, in sebocytes; the inhibition of testosterone’s gonadal production; decreasing free testosterone by increasing the production of sex hormone-binding globulin (SHBG); and downregulating the genes involved in sebaceous glands’ growth and production of lipids [6,44]. Several studies indicate that estrogens have anti-inflammatory roles in acne events; in particular, 17β-Estradiol inhibits interferon-γ in human keratinocytes [53]; and estrogens block proinflammatory cytokines (IL-1, IL-6, and TNF-α) and NF-κB [54,55], and repress macrophage activation and monocyte recruitment in inflammatory occasions [56,57,58].

### 3.2. Seborrhoea

The typical regions of the skin that are affected by acne are the face and upper torso, as they are densely populated by sebaceous glands [3]. Those glands are cutaneous appendages that secrete an oily substance (sebum) within hair follicles through sebaceous ducts [59]. Sebum is a mixture of lipids—namely a combination of wax esters, triglycerides, steroid esters, and squalene [60]—protecting the skin against friction due to natural lubrication; by acting as a barrier, it retains water molecules helping to keep the skin moist and healthy [42]. However, hyperseborrhea, excessive sebum production associated with overactive sebaceous glands, is a major etiopathogenetic factor directly associated with AV, because it contributes to all three mechanisms described below—comedogenesis, microbial proliferation, and inflammation—whereas the disease is not seen in the absence of sebum [4].

It is possible that not only the amount of sebum is a key factor in acne; the alteration of sebum composition may also have a determinant role. For instance, lipidic fractions of sebum are proinflammatory, contributing to the inflammatory cell and tissue process, ultimately resulting in the development of acne lesions [60,61]. For example, squalene peroxide induces inflammatory responses in keratinocytes by increasing IL-6 production and lipoxygenase (5-LOX) activation [62]. Moreover, modifications in the sebum ratio of saturated/unsaturated fatty acid are triggers of innate immunity response and follicular inflammation, being a main character in early inflammatory pathways of acne [47,60]. Additionally, changes in the oxidant/antioxidant ratio also represent a cause for acne’s initial development [32]. Further acne-related lipid-enzymes are liver X receptor-α (LXR-α) and cyclooxygenase 2 (COX-2), which regulate inflammation and lipid synthesis. LXR-α is expressed in sebaceous glands, sweat glands, and hair follicles, and controls the transcription of the genes involved in fatty acid and lipid synthesis. During inflammatory events, COX2 release is stimulated by cytokines that were activated by NF-κB [63].

### 3.3. Comedogenesis

Increased androgen production, the accumulation of sebum, and the adhesiveness of keratinocytes lead to follicle blockage and the occlusion of the pilosebaceous ducts, resulting in the formation of microcomedones that are the primary type of acne lesions [7,64]. These hard structures, microcomedones, are formed within the hair follicle, more precisely in the infundibulum, and are connected with the sebaceous gland via a keratinized duct. The set of the hair follicle and sebaceous gland constitutes the pilosebaceous unit [59]. As a pleomorphic disorder of the pilosebaceous unit, acne microcomedones evolve into comedones owing to sebum and keratin accumulation accompanied by follicle expansion [58], and these can be closed (white heads) or open (blackheads), but they may progress to inflammatory lesions, such as pustules, papules, cysts, and nodules [30], eventually leaving scars [6].

Keratinocyte IL-1 secretion is another triggering step in the comedogenesis process, mostly as a consequence of *C. acnes*-mediated Toll-like receptor (TLR) activation. IL-1 also contributes to sebocytes hypercornification. Comedone formation is a result of a combination of some factors, such as the response to androgen production; sebum alteration, production, and oxidation; *C. acnes* proliferation, and recruitment of cytokines to the pilosebaceous unit [7].

### 3.4. Microbial Proliferation

The skin microbiome is composed of commensal microbes of which the ultimate function is to maintain skin homeostasis, representing the resident microbiome (usually harmless and non-pathogenic), but also of other microbes that commonly are pathogenic and temporary, representing the transient microbiome. Despite that, the microbiome at AV lesions is usually constituted by *C. acnes* and *Staphylococcus epidermidis,* which together set the resident microbiome, while the transient pathogen is typically *Staphylococcus aureus* [65,66]. Although the resident microbes are usually non-pathogenic, it is accepted that both *C. acnes* and *S. epidermidis*—in an acne context—become pathogenic, either through hyperproliferation or by other mechanisms [65,67]. On the other hand, though they are commonly called “acne-related bacteria”, new insights show that *S. epidermidis* has a beneficial role in acne through an anti-*C. acnes* action when controlling *C. acnes* hyperproliferation, and by suppressing *C. acnes*-induced IL-6 and TNF-α production in keratinocytes [65,67]. The activation of TNF-α stimulates lipogenesis through JNK (c-Jun N-terminal kinase), a family member of the MAPK, PI3K (phosphoinositide 3-kinase), and protein kinase B (PKB) pathways [38]. While this information is available now, in recent past years *S. epidermidis’* role was a controversial topic in which the discussion relied on whether it may become pathogenic in an acne context or not, being considered a target which needed to be eliminated in an acne context by some researchers, possibly supported by being the most frequent cause of nosocomial infections, having immune invasion capacity, and being a producer of lipolytic enzymes that may damage follicles, leading to comedogenesis [67,68].

*Cutibacterium acnes* is the main bacterium linked to acne pathogenesis and is one of the main triggers associated with the disease [4]. It is a Gram-positive, anaerobic, and lipophilic diphtheroid. Commensal, and an abundant member of the skin microbiota [69], the bacterium’s natural habitat is the pilosebaceous follicles, and in regular, non-pathogenic situations, this diphtheroid helps to maintain the homeostasis of a healthy skin [34]. Nevertheless, this bacterium can induce the production of proinflammatory molecules while converting triglycerides (from sebum) into free fatty acids [7]. Its pathogenic features also include infectious mechanisms, such as chemo-attractive mediators’ production, biofilm formation, and the release of virulence factors that mediate the immune response, among others [35,69]. The hyperproliferation of *C. acnes* is another factor of AV due to the secretion of propionic acid that influences the formation of comedones, which ultimately leads to keratinocyte differentiation [69]. *Cutibacterium acnes* creates a biofilm by attaching to and growing on the surface of corneocytes, producing extracellular polymers contributing to the process of microcomedones formation [33]. Furthermore, *C. acnes* is capable of activating several pathways leading to the release of cytokines (IL-1, IL-6, IL-8, IL-10, IL-12, and TNF-α) that activate the immune system to produce inflammation via different pathways, including Toll-like receptors that induce the liberation of antimicrobial peptides (human β-defensin-1 and human β-defensin-2) and trigger matrix metalloproteinases, lipases, proteinases, and hyaluronidases backing dermal matrix destruction, which together lead to acne inflammation and the formation of scars [32,70]. A change of paradigm has come from recent research that points out a specific influence of *C. acnes* in acne lesions. Not the number of bacterial cells is important, but instead the loss of balance between diverse *C. acnes* phylotypes that trigger the immune system activation, consequently undergoing inflammation, particularly due to a predominance of *C. acnes* phylotype IA1 [65].

### 3.5. Inflammatory Response

The AV pathophysiological cascade ends with the inflammatory process, but, at the same time, it also follows the whole process from the beginning, by activating the immune system and/or by the release of molecules that activate inflammatory pathways [71]. There are many inflammatory mediators involved, as mentioned before, e.g., proinflammatory lipids (at the hyperseborrhea process), cytokines (mostly involved by recruitment of *C. acnes*), and chemokines (stimulated by bacterial antigens). There is also the role of proinflammatory cathelicidins, peptides from macrophage lysosomes, and members of the immune system with immunomodulatory and antimicrobial functions [7].

It seems that the main factor influencing the inflammatory process is the activation of the immune system by *C. acnes*, when the bacterium overpopulates sebocytes, through TLRs and nod-like receptors (NLRs) that segregate IL-1 and other cytokines, activating inflammatory pathways [7]. Furthermore, the keratinocyte stimulated-*C. acnes* production of reactive oxygen species (ROS) induces nitric-oxide production in macrophages, contributing to the magnification of the inflammatory response [69].

## 4. Seaweed Extracts and Compounds to Address AV Disease

Considering the stated pathophysiological mechanisms of AV, this work will further address the potential uses of red macroalgae against all five of the therapeutic targets described above. The first three processes listed (Section 3.1 hormonal influence, Section 3.2 seborrhoea, Section 3.3 comedogenesis) are harder to analyze in vitro, and this is reflected in the much smaller number of studies targeting these processes. However, numerous studies have identified red seaweeds as significant producers of valuable metabolites with antibacterial and anti-inflammatory activities, thus representing a potential resource in the battle against AV, a chronic disease of which the management involves the mitigation of these symptoms.

### 4.1. Antibacterials from Red Macroalgae

The antibacterial activity of red seaweed extracts and/or compounds against *C. acnes* and *S. epidermidis* is summarized in Table 1. Keeping in mind that the role of *S. epidermidis* is still controversial in acne pathology, a literature revision about compounds and extracts from red seaweeds that inhibit the growth of this bacterium was included in this table. However, one should acknowledge that these studies were bioprospecting natural products against this species, and therefore generating the presented data, whereas nowadays, given the recent discovery of a positive role of *S. epidermidis* as an anti-*C. acnes* agent, it might be more interesting to find the extracts that do not inhibit this species, or that do so to a lower extent than against *C. acnes*.

*Staphylococcus aureus* is a common human pathogen which has also been associated with AV pathology on several occasions [72]. There are plenty of research articles proving that red seaweeds are an excellent source of anti-*S. aureus* compounds, exploiting the potential of, for example, *Laurencia johnstonii*, *Laurencia obtusa* var. *pyramidata*, *Gracilaria dendroides*, *Grateloupia turuturu*, *Corallina officinalis*, and *Rissoella verruculosa* [73,74,75,76,77,78,79,80]. Such studies were not included in the summarized Table 1, because that would result in hundreds of entries; this has been reviewed quite recently [81].

Research concerning bioactives to inhibit the growth of *C. acnes* and *S. epidermidis* resulted in the study of 28 different species of red seaweeds. Though Rhodophyta is the largest group of macroalgae, including about 7000 species [82], the fact that only 28 of them have been studied for this purpose shows that there is still room to proceed with studies to discover antibacterials from this macroalgae which are applicable in AV (specific towards *C. acnes*).

When it comes to *C. acnes*, there is a great lack of knowledge, wherein only five studies have approached the antibacterial capacity of 11 red macroalgal species but, notwithstanding, reported results show great capacity of growth inhibition. One example is a methanol crude extract of *Symphyocladia latiuscula*, having an MIC (minimal inhibitory concentration) of 0.16 mg·mL^−1^ [83] for a compound isolated from *Osmundaria serrata*, lanosol ethyl ether, which showed MIC at 0.08 mg·mL^−1^ and MBC (minimal bactericidal concentration) at 0.5 mg·mL^−1^ [23]. The study by Choi and co-workers [83] employed methanol solid–liquid extractions from *S. latiuscula*, resulting in an extract capable of inhibiting *C. acnes* growth. This seaweed is known to have high amounts of bromophenols, which can explain the antibacterial effect, because these compounds are known to be toxic to some bacteria [83]. The work of Barreto and Meyer (2006) aimed to isolate lanosol ethyl ether from *O. serrata*, which is a brominated phenol and a halogenated metabolite, showing high bacteriostatic and mild bactericidal activity [23]. Organobromine compounds are naturally produced by seaweeds for chemical defense and are usually found in Rhodophyta. Besides lanosol, examples include acetogenins (brominated nonterpenoid metabolites), which are mainly found in the genus *Laurencia* [84]; bromoform from *Asparagopsis taxiformis* [85]; brominated monoterpenes, also in the genus *Laurencia* [86], but also in *Plocamium cartilagineum* [84]; and indoles from *Rhodophyllis membranacea* [84]. These bromine compounds confer several bioactivities, including antimicrobial, antioxidant, anticancerogenic, and anti-diabetic activities [87], emphasizing the high value of red seaweed-derived compounds. Another example showing the antibacterial activity conferred by organobromine compounds is the study by [88], which used *Asparagopsis armata* supercritical CO_2_ extractions to find an inhibition halo of 23 mm (at 10 mg·mL^−1^) in contact with a *C. acnes* culture. Such results seem to be in accordance with a variety of cosmeceutical products which recently became available in the market that incorporate *A. armata* extracts. For instance, ASPAR’AGE™, formulated by SEPPIC (La Garenne-Colombes, France), promises a lotion with an extract containing MAAs that reduce the visible effects of age, and Asparcid P^®^, from Exsymol (Monaco-Ville, Monaco), claims to possess cytostimulating action and antimicrobial activity.

Regarding *S. epidermidis*, there are few studies searching antibacterials from 19 different red macroalgae, probably because this bacterium is a pathogen when the immune system is compromised and is not only involved in acne pathophysiology but also, above all, a relevant matter of concern when it comes to severe hospital-acquired infections from decades ago [89], wherein the research material is more abundant, but unrelated to the present work. A study of 1991 showed an inhibitory halo of 7.5 mm when a piece (3 mm) of *Polysiphonia fibrillosa* was in contact with an *S. epidermidis* culture [90]. It is possible that this antibacterial activity may be due to bromophenolic compounds, because other studies reported those properties in the extracts of other species belonging to the genus *Polysiphonia*, namely a *Polysiphonia decipiens* compound that was a mild growth inhibitor towards *S. aureus*, *Bacillus subtilis*, *Micrococcus luteus*, *Proteus vulgaris*, and *Salmonella typhimurium* [91], and *Polysiphonia denudata* and *Polysiphonia denudata* f. *fragilis* effective against *S. aureus* and *Escherichia coli* [92]. More recently, a bromophenol (bis (2,3-dibromo-4,5-dihydroxybenzyl) ether) isolated from *Rhodomela confervoides* showed MIC at 35 µg·mL^−1^ [93] exhibiting the highest antibacterial activity (Table 1). The *Rhodomela* genus is another example containing high levels of brominated compounds—in this case, a bromophenol—and the first two marine dibromophenols were isolated from this genus in 1967, more specifically, from the red algae *Rhodomela larix* [94].

Different extracts and compounds can be obtained or isolated by using several solvents of extraction and/or isolation procedures. So far, the most-used solvents to obtain crude extracts and isolated compounds with antibacterial properties, when facing the growth of *C. acnes* or *S. epidermidis*, are methanol, ether, acetone, toluene, chloroform, and hexane (Table 1). Given the limited variety of red macroalgae explored for this purpose, according to the research, the genus *Laurencia* was the most often used in crude extracts. *Laurencia* species have been studied for years, revealing this to be a very rich genus in bioactive metabolites, such as diterpenes, triterpenes, sesquiterpenes, steroids, aromatic compounds, and indoles, among others [95], and sesquiterpenoids are the main constituents from this genus [96]; among other properties, they are known to possess potent antibacterial activities against methicillin-resistant *S. aureus* (MRSA), penicillin-resistant *Streptococcus pneumoniae*, and vancomycin-resistant *Enterococcus faecalis* [97]. From a crude methanol extract of *R. confervoides*, ten bromophenols were isolated, confirming the reputation of the richness in bromophenol metabolites of the genus *Rhodomela* [93,94].

Oddly, no work with aqueous extracts addressing the discovery of antibacterials, regarding *C. acnes* and *S. epidermidis*, was found. This type of extraction, besides being industry-friendly [98], is a method of obtaining antibacterial bioactives from Rhodophyta, as it allows the recovery of polysaccharides and MAAS, for example, that successfully inhibit *E. coli*, *S. aureus*, multidrug-resistant *Salmonella*, and *Vibrio harveyi* [77,99]. Another industry- and environmentally friendly way to obtain extracts is using ethanol as extracting solvent, or even by obtaining essential oils, a technique known as solvent-free extraction. Ethanolic extracts from *Gracilaria textorii*, *Gracilaria verrucosa*, *Grateloupia angusta*, *Grateloupia crispata*, *Grateloupia elliptica*, and *Meristotheca papulose* all showed *C. acnes* growth inhibition, in a study by Lee et al. [100] aiming to find anti-*C. acnes* capacity in Korean edible seaweeds. Ethanolic extractions can retrieve, among others, phenolic compounds, sterols, and carotenoids [98], and the antibacterial properties of these have been attested [24,101,102]. Essential oil with anti-*S. epidermis* activity was obtained from *L. obtusa* [78], corroborating the renowned antimicrobial properties of essential oils obtained from Rhodophyta [103,104,105].

Table 1’s outcomes reveal that both the red seaweeds extracts and the compounds have antibacterial activity against acne-related bacteria. Despite this, there is still plenty of room to study a wide variety of Rhodophyta species, using extractions with different solvents, and isolating its compounds, in order to obtain a more diverse range of compounds and assess their antibacterial potential in an AV context. Furthermore, it is important to understand how different compounds could interfere in the inhibition of bacterial growth, and to study their mechanisms of action. Beyond this, it is also crucial to analyze further how the skin microbiome would be affected by the compounds and, ultimately, to perform in vivo studies to prove compounds’ efficiency.

**Table 1:** Antibacterial compounds and extracts derived from red seaweeds which are effective on *Cutibacterium acnes* and *Staphylococcus epidermidis* growth. SLE: solid–liquid extraction; UE: ultrasonic extraction; SM: steam distillation; MeOH: methanol; MIC: minimum inhibitory concentration; MBC: minimal bactericidal concentration; IC50: half-maximal inhibitory concentration; * *Staphylococcus epidermidis* clinical isolate; NA: not applicable.

Red Macroalgal Species	Compound or Extract (Technique)	Concentration/ Volume Tested	Antibacterial Assay	Outcome	Reference
*Cutibacterium acnes*					
*Asparagopsis armata*	Supercritical extract	5 mg·mL^−1^	Disc Diffusion	Zone of inhibition 17.33 ± 0.58 mm	[88]
	Supercritical extract	10 mg·mL^−1^	Disc Diffusion	Zone of inhibition 23.00 ± 1.00 mm	[88]
*Gracilaria textorii*	EtOH (UE)	0–1.024 μg·mL^−1^	MIC	Inhibitory concentration > 1.024 μg·mL^−1^	[100]
*Gracilaria verrucosa*	EtOH (UE)	0–1.024 μg·mL^−1^	MIC	Inhibitory concentration > 1.024 μg·mL^−1^	[100]
*Grateloupia angusta*	EtOH (UE)	0–1.024 μg·mL^−1^	MIC	Inhibitory concentration > 512 μg·mL^−1^	[100]
*Grateloupia crispata*	EtOH (UE)	0–1.024 μg·mL^−1^	MIC	Inhibitory concentration > 512 μg·mL^−1^	[100]
*Grateloupia elliptica*	EtOH (UE)	0–1.024 μg·mL^−1^	MIC	Inhibitory concentration > 512 μg·mL^−1^	[100]
*Meristotheca papulosa*	EtOH (UE)	0–1.024 μg·mL^−1^	MIC	Inhibitory concentration > 256 μg·mL^−1^	[100]
*Osmundaria serrata*	Lanosol ethyl ether	25 to 0.01 mg·mL^−1^	MBC	Bactericidal concentration 0.50 ± 0.29 mg·mL^−1^	[23]
	Lanosol ethyl ether	25 to 0.01 mg·mL^−1^	MIC	Inhibitory concentration 0.08 ± 0.02 mg·mL^−1^	[23]
*Plocamium telfairiae*	EtOH (UE)	0–1.024 μg·mL^−1^	MIC	Inhibitory concentration > 256 μg·mL^−1^	[88]
*Sphaerococcus coronopifolius*	12S-hydroxy-bromosphaerol	0.1–200 µM	IC50	Inhibitory concentration 10.88 (7.83–15.12) µM	[106]
	12R-hydroxy-bromosphaerol	0.1–200 µM	IC50	Inhibitory concentration 8.75 (6.51–11.77) µM	[106]
	Bromosphaerol	0.1–200 µM	IC50	Inhibitory concentration 14.06 (10.41–19.00) µM	[106]
*Symphyocladia latiuscula*	MeOH (SLE)	1 mg·disc^−1^	Disc Diffusion	Zone of inhibition 3.5 ± 1.3 mm	[83]
	MeOH (SLE)	5 mg·disc^−1^	Disc Diffusion	Zone of inhibition 8.8 ± 0.8 mm	[83]
	MeOH (SLE)	19.5 μg–10 mg·mL^−1^	MIC	Inhibitory concentration 0.16 mg·mL^−1^	[83]
*Staphylococcus epidermidis*					
*Asparagopsis taxiformis* (Falkenbergia-phase)	MeOH (SLE)	120 µL	Well Diffusion	Zone of inhibition 21 ± 2.31 mm	[107]
*Bryothamnion seaforthii*	Lectin	125; 250 μg·mL^−1^	Microdilution (Growth Inhib)	Inhibitory concentration 10–40%	[108]
*Chondrus crispus*	NA	NA	Piece of algae (3 cm)	Zone of inhibition 6.8 mm	[90]
	MeOH (SLE)	100 µ (of 4 mg·mL^−1^)	Disc Diffusion	Zone of inhibition 21 mm	[90]
*Cystoclonium purpureum*	NA	NA	Piece of algae (3 cm)	Zone of inhibition 8.2 mm	[90]
*Gracilaria corticata*	MeOH:toluene 3:1 (SLE)	120 µL	Well Diffusion	Zone of inhibition ± 4 mm	[109]
*Gracillaria gracilis*	Diethyl ether (SLE)	25 µL	Disc Diffusion	Zone of inhibition 15 mm	[110]
*Grinnellia americana*	NA	NA	Piece of algae (3 cm)	Zone of inhibition 7 mm	[90]
*Hypnea musciformis*	Lectin	250 μg·mL^−1^	Microdilution (Growth Inhib)	Inhibitory concentration 10–40%	[108]
*Hypnea pannosa*	MeOH:toluene 3:1 (SLE)	120 µL	Well Diffusion	Zone of inhibition ± 7.5 mm	[109]
*Jania rubens*	MeOH (SLE)	2 mg·disc^−1^	Disc Diffusion	Zone of inhibition 6.5 mm	[111]
	MeOH (SLE)	4 mg·disc^−1^	Disc Diffusion	Zone of inhibition 11 mm	[111]
	Chloroform (SLE)	4 mg·disc^−1^	Disc Diffusion	Zone of inhibition 10 mm	[111]
*Laurencia majuscula*	Elatol	30 mg·disc^−1^	Disc Diffusion	Zone of inhibition 19–24 mm *	[112]
	Elatol	1–4 mg·mL^−1^	MIC	Inhibitory concentration 2 mg·mL^−1^ *	[112]
*Laurencia obtusa*	Essential oil	0.1 µL	Disc Diffusion	Zone of inhibition 9 mm	[78]
	Essential oil	0.2 µL	Disc Diffusion	Zone of inhibition 10 mm	[78]
	Essential oil	0.4 µL	Disc Diffusion	Zone of inhibition 11 mm	[78]
	Chloroform (SLE)	2 mg·disc^−1^	Disc Diffusion	Zone of inhibition 6.5 mm	[78]
	Hexane (SLE)	2 mg·disc^−1^	Disc Diffusion	Zone of inhibition 6.5 mm	[78]
*Laurencia obtusa* var. *pyramidata*	Chloroform (SLE)	2 mg·disc^−1^	Disc Diffusion	Zone of inhibition 6.5 mm	[78]
	Hexane (SLE)	2 mg·disc^−1^	Disc Diffusion	Zone of inhibition 6.5 mm	[78]
*Laurencia* sp.	MeOH (SLE)	120 µL	Well Diffusion	Zone of inhibition 11 ± 2.56 mm	[107]
*Plocamium angustum*	Costatone C	0.5–129 µg·mL^−1^	MIC	Inhibitory concentration 64 µM	[113]
*Polysiphonia fibrillosa*	NA	NA	Piece of algae (3 cm)	Zone of inhibition 7.5 mm	[90]
*Rhodomela confervoides*	3-bromo-4-[2,3-dibromo-4,5-dihydroxyphenyl] methyl-5-(hydroxymethyl) 1,2-benzenediol	35–140 μg·mL^−1^	Microdilution (MIC)	Inhibitory concentration 140 μg·mL^−1^	[93]
	3-bromo-4-[2,3-dibromo-4,5-dihydroxyphenyl] methyl-5-(hydroxymethyl) 1,2-benzenediol	35–140 μg·mL^−1^	Microdilution (MIC)	Inhibitory concentration >140 μg·mL^−1^ *	[93]
	3-bromo-4-[2,3-dibromo-4,5-dihydroxyphenyl] methyl-5-(ethoxymethyl) 1,2-benzenediol	35–140 μg·mL^−1^	Microdilution (MIC)	Inhibitory concentration 140 μg·mL^−1^	[93]
	3-bromo-4-[2,3-dibromo-4,5-dihydroxyphenyl] methyl-5-(ethoxymethyl) 1,2-benzenediol	35–140 μg·mL^−1^	Microdilution (MIC)	Inhibitory concentration 140 μg·mL^−1 *^	[93]
	3-bromo-4-[2,3-dibromo-4,5-dihydroxyphenyl] methyl-5-(methoxymethyl) 1,2-benzenediol	35–140 μg·mL^−1^	Microdilution (MIC)	Inhibitory concentration 70 μg·mL^−1^	[93]
	3-bromo-4-[2,3-dibromo-4,5-dihydroxyphenyl] methyl-5-(methoxymethyl) 1,2-benzenediol	35–140 μg·mL^−1^	Microdilution (MIC)	Inhibitory concentration 70 μg·mL^−1 *^	[93]
	4,40-methylenebis [5,6-dibromo-1,2-benzenediol]	35–140 μg·mL^−1^	Microdilution (MIC)	Inhibitory concentration 140 μg·mL^1^	[93]
	4,40-methylenebis [5,6-dibromo-1,2-benzenediol]	35–140 μg·mL^−1^	Microdilution (MIC)	Inhibitory concentration 140 μg·mL^−1^ *	[93]
	bis (2,3-dibromo-4,5-dihydroxybenzyl)ether	35–140 μg·mL^−1^	Microdilution (MIC)	Inhibitory concentration 35 μg·mL^−1^	[93]
	bis (2,3-dibromo-4,5-dihydroxybenzyl)ether	35–140 μg·mL^−1^	Microdilution (MIC)	Inhibitory concentration 140 μg·mL^−1^ *	[93]
*Sphaerococcus coronopifolius*	12S-hydroxy-bromosphaerol	0.1–200 µM	Microdilution (IC50)	Inhibitory concentration 10.07 (7.84–12.94) µM	[106]
	Sphaerococcenol A	0.1–200 µM	Microdilution (IC50)	Inhibitory concentration 56.58 (41.01–78.06) µM	[106]
	12R-hydroxy-bromosphaerol	0.1–200 µM	Microdilution (IC50)	Inhibitory concentration 5.61 (4.18–7.53) µM	[106]
	Bromosphaerol	0.1–200 µM	Microdilution (IC50)	Inhibitory concentration 9.05 (7.05–11.63) µM	[106]
*Symphyocladia latiuscula*	MeOH (SLE)	19.5 μg·mL^−1^–10 mg·mL^−1^	MIC	Inhibitory concentration 0.63 mg·mL^−1^	[83]

### 4.2. Anti-Inflammatory Extracts and Compounds from Red Macroalgae

The literature revision regarding both anti-inflammatory compounds and extracts from Rhodophyta resulted in 75 research articles, which are summarized in Table 2. These papers included 56 different red macroalgal species, wherein the *Gracilaria* genus was the most abundant, resulting in 31 outcomes. Regarding species, *G. verrucosa*, *Porphyra dentata*, and *Pyropia yezoensis* had the highest scores, presenting 14, 10, and 9 results, respectively.

As mentioned across Section 3, inflammatory responses occur during all of the stages of AV lesions’ formation. These include increasing androgen production that leads to the higher activity, size, and enlargement of the sebaceous glands, consequently producing more sebum; increased lipid synthesis due to LXR-α, COX2, and NF-κB action; inflammatory sebum lipid fractions; the increased segregation of growth hormones, which stimulates the production of IGF’s, consequently activating MAPK/ERK pathways; and *C. acnes* activation of a vast array of cytokines (IL-1, IL-6, IL-8, IL-10, IL-12 and TNF-α), TLR’s, and β-defensins. Relevant studies targeting some of these inflammatory markers are included in Table 2 and are detailed hereafter.

Rhodophyta species are known for the rich composition in polysaccharides of their cell walls and intercellular matrix. These include non-sulphated polysaccharides (agars and carrageenans), sulphated galactans, and neutral-structural polysaccharides (cellulose, mannans, and xylans) [15]. The analyses of Table 2 reveal that polysaccharides are the compounds that appear in greater quantity, representing the greater number of studies that have reported anti-inflammatory compounds from red seaweed. Isolated compounds classified as polysaccharides by the authors revealed several anti-inflammatory properties, namely the degranulation of basophils inhibition (*Chondrus verrucosus* ([114]), the reduction of paw edema (*Porphyra vietnamensis* [115], *Digenia simplex* [116]) and leukocyte infiltrations (*D. simplex* [116]), the inhibition of IL-1β, TNF-α (*D. simplex* [116]; *Gracilaria lemaneiformis* [117]), NO, PGE2, COX-2 and ROS (*Gracilaria lemaneiformis* [117]) production, and NF-κB expression (*Porphyridium* sp. [118]). Furthermore, cellulose microfibril from *Gelidium amansii* lead to JNK1/2 and p38 inhibition [119], and cellulose nanocrystals from the same seaweed revealed COX-2 and ERK1/2 inhibition [120]. Sulphated polysaccharide outcomes include reduced neutrophil (*Agardhiella ramosossima* [121]) and leukocyte (*Gracilaria birdae* [122], *Gracilaria caudata* [123], *Gracilaria cornea* [124], *Hypnea musciformis* [125]) migration/infiltration; reduced paw oedema (*Agardhiella ramosossima* [121], *Gelidium crinale* [126], *Gracilaria birdiae* [122], *Gracilaria caudata* [123], *Gracilaria cornea* [124] *Hypnea musciformis* [125]); NO and TLR4 inhibition (*Gelidium pacificum* [127]); IL-1β and TNF-α inhibition (*Gracilaria caudata* [123], *Solieria filiformis* [128]); and COX-2 and 5-LOX inhibition (*Gracilaria opuntia*, *Kappaphycus alvarezii* [129]). These studies included red seaweed compounds which were able to inhibit ILs and TNF-α, which are important triggers of the inflammatory pathways involved in AV, among others. These markers of inflammation are able to activate macrophages that excessively induce the release of pro-inflammatory cytokines and other mediators of inflammation, wherein PGE2 is an inflammatory mediator generated by the COX2 conversion of arachidonic acid [130], NO is a known endogenous free radical and a pro-inflammatory mediator [131], interleukins induce the synthesis of acute phase proteins [132], and TNF-α is one of the most important pro-inflammatory cytokines that participates in vasodilatation, edema formation, and the expression of adhesion molecules that leads to leukocyte adhesion to epithelium [133]. In fact, species belonging to the genera *Gracilaria* and *Gelidium* seem to be a good source of compounds, in particular sulphated polysaccharides and agaro-oligosaccharides (non-sulphated polysaccharides), which resulted in ILs and TNF-α inhibition. Specific examples include *Gracilaria lemaneiformis* isolated agaro-oligosaccharides evaluated in LPS-stimulated RAW264.7 [117] that inhibited several inflammatory triggers, as did sulphated galactan from *Kappaphycus alvarezii* [129]. Furthermore, porphyran extracted from *P. yezoensis* supressed IL-6, IL-12, and TNF-α expression in the lipopolysaccharide-induced activation of dendritic cells in mice [134]. Sulphated galactans from both red algae possess anti-inflammatory activity due to the suppression of COX-2 and 5-LOX, and, in accordance with the study of de Sousa et al. [126], the same type of compounds isolated from *Gelidium crinale* reduced paw oedema on rat-stimulated paw oedema. Curiously, sulphated polysaccharides, such as carrageenan from red seaweed, have been employed for decades to induce local inflammation in in vivo studies using rats, by stimulation through TLR4 signalling pathways [135,136], as a study using kappa/beta-carrageenan proved in human blood cells by the increase of the IL-10 marker [137]. Thus, cases reported where sulphated galactans are anti-inflammatory lead to the conclusion that the subtle differences in the chemical structure of sulphated polysaccharides, among red macroalgal species, profoundly impact their immunomodulatory properties.

Seaweeds usually contain a low quantity of lipids, but despite that, they synthesize important ones, such as omega-3 and -6, unsaturated fatty acids (UFA), polyunsaturated fatty acids (PUFA), highly unsaturated fatty acids (HUFA), and monounsaturated fatty acids (MUFA) [138]. Two studies reported lipid compounds that showed anti-inflammatory activities (Table 2). For example, in lipopolysaccharide (LPS)-stimulated mouse macrophage cell line RAW 264.7 combined with *Gracilaria verrucosa*, Hung et al. determined that there was significant reduction of NO, TNF-α and IL-6 with the 14 lipid compounds (20 µg·mL^−1^), including prostaglandins, ceramides, fatty acids, and oxygenated fatty acids [139]. These pro-inflammatory mediators were successfully inhibited by the positive influence of the algal compounds and extracts, confirming their anti-inflammatory properties in the most-used cell line when it comes to biological inflammatory assays [140].

Red macroalgae are a source of metabolites, including halogenated compounds, such as terpenoids and derivatives [141]. Studies show that isolated diterpenes, diterpenoids, and meroterpenoids have anti-inflammatory properties (Table 2). It should be noted that COX-2 and 5-LOX are fundamental enzymes involved in lipid synthesis and are therefore triggers on the AV cascade. Once again, three *Gracilaria* species showed promising results, in this case, by the inhibition of both enzymes. The newly described salicornolides (A, B, and C) from *G. salicornia* successfully inhibited both anti-inflammatory markers [142], in the same way that three compounds from the same algae did, which were classified as a one diterpenoid and two 2H-chromenyl derivatives [143]. Similarly, three different compounds—namely an oxygenated meroterpenoid (from *Kappaphycus alvarezii* [144]), a methylfuran derivative (*Gracilaria opuntia* [145]), and a halogen derivative [146]—were able to inhibit both enzymes, except for the methylfuran derivative, which only inhibited 5-LOX [145]. Furthermore, a diterpene from *Laurencia glandulifera,* classified as Neorogioltriol, showed the ability to inhibit not only COX-2, but also NO, TNF-α, NF-κB, and the reduction of paw edema [141].

Seaweeds also synthesize phenolic compounds including phlorotannins, bromophenols, and mycosporine-like amino acids (MAAs) [12]. Found especially in Rhodophyta species are bromophenol, flavonoids, bromated compounds, and MAAs, as they also present anti-inflammatory activities (Table 2). Nuclear factor NF-κB is, perhaps, the most significant transcription factor associated with AV, because it transcribes numerous inflammatory genes, including TNF-α, IL-1, IL-6, and IL-8 [147,148,149]. Porphyra species seem to be an excellent source of metabolites which are able to inhibit the transcription factor. For example, a study by Kazłowska et al. [150] demonstrated that a methanol extract, catechol, and rutin (flavonoid), from *P. dentata*, were able to inhibit NF-κB, and these two phenolic compounds additionally inhibited iNOS and reduced NO levels. A report on Porphyra-334 also showed NF-κB inhibition in an LPS-challenged RAW264.7 cell line [151]. Likewise, two studies using *P. yezoensis* showed similar results in other cell lines. *P. yezoensis* aqueous protein extract downregulated MAPK and NF-κB in the HK2 human proximal tubular epithelial cell line [152]; extracted MAAs obtained through an ethanol extraction, and reduced the expression of IL-1β, IL-6, IL-10, NF-κB in the skin of male ICR mice [153]. Other species can be included as containers of compounds which are able to inhibit NF-κB, including *G. lanceolata* [154], *L. glandulifera* [141], *Polysiphonia morrowii* [155], and *Pyropia yezoensis* [156]. In this latter study, the use of methanol extracts from *P. yezoensis* revealed TARC (thymus and activation-regulated chemokine) and MDC (macrophage-derived chemokine) expression inhibition, ERK (extracellular signal-regulated kinase), JNK (c-Jun N-terminal kinase), p38 inhibition, and nuclear factors NF-κB and IkB-α downregulation, when using the cell line HaCaT induced with interferon (IFN)-ϒ and HaCaT induced with TNF-α [156]. HaCaT are immortalized human skin keratinocytes that produce pro-inflammatory cytokines affecting the development of inflammatory skin disorders [119]. *Pyropia yezoensis* is rich in bioactive compounds such as amino acids, polysaccharides, phytosterols, and pigments. The study by Ha et al. [156] in keratinocytes, using that macroalgae, vouched for the anti-inflammatory activity of its extracts by suppressing pro-inflammatory chemokines. Although MAAs are presented here as anti-inflammatory agents, in accordance with most of the studies we found, a report by Becker et al., (2016) proved that not all MAAs are anti-inflammatory; some are pro-inflammatory, even when the chemical structure is similar. This is the case of shinorine and porphyra-334 isolated from *P. yeozensis*, where porphyra-334 inhibited NF-κB and supressed IDO-1, and on the contrary, when using shinorine in LPS-stimulated RAW264.7, the result was the increase of NF-κB and the suppression of IDO-1 [151].

Other algae species that should be highlighted are *Palmaria palmata*, *Pyropia yezoensis*, and *Laurencia okamurae*. *Palmaria palmata* thermolysin-digested water extract reduced NO, TNF-α, and IL-6 on LPS-stimulated RAW264.7, and also reduced the paw oedema of rat carrageenan-induced paw oedema [157]; the seaweeds’ phenolic extract reduced ROS, NO, MPO, IL-8, IL-1Β, IL-6, TNF-α, and downregulated TLR4 on LPS-stimulated primary human neutrophils cell line [158]; the lipid extract of the same algae downregulated the expression of 14 pro-inflammatory genes (TLR1, TLR2, TLR4, TLR8, TRAF5, TRAF6, TNFSF18, IL6R, IL23, CCR1, CCR4, CCL17, STAT3, and MAP3K1) in LPS-stimulated Human THP-1 macrophages [159]. *Pyropia yezoensis* isolated peptide PPY1 inhibited NO, ROS, iNOS, COX-2, IL-1Β, and TNF-α, and downregulated p38 and MAPK in LPS-stimulated RAW264.7 [160]. A strong activity was also found in an ethyl acetate extract from *L. okamurae* at 25 µg·mL^−1^, using LPS-stimulated RAW264.7, inhibiting the production of NO, prostaglandin E2 (PGE2), IL-6, and TNF-α [161].

The final highlight of the results in Table 2 should be given to a relevant number of studies that were condcted in vivo using mice and rats. Most of the reports described the reduction of paw oedema [115,121,126,141,157,162,163,164,165,166,167] and reduced leukocyte influx/migration [168], or even both [116,122,123,124,125,169,170], proving that red seaweed-derived compounds are effectively anti-inflammatory. Though a significant number of compounds isolated from Rhodophyta have proven to be anti-inflammatory, both in vitro and in vivo, the opportunity to investigate how these compounds behave in individuals suffering from AV remains open. Successful anti-inflammatory in vitro studies on inflammatory markers involved in the AV cascade show a promising potential for human clinical trials.

**Table 2:** Anti-inflammatory compounds and extracts derived from red seaweeds which are effective on different inflammation models. SLE: solid–liquid extraction; SPE: solid-phase extraction; MeOH: methanol; HaCat: immortalized human skin keratinocyte cell line; RAW264.7: macrophage cell line; HK2: human proximal tubular epithelial cell line; NO: nitric oxide; JNK: c-Jun N-terminal kinase; IL: interleukin; TNF: tumor necrosis factor; iNOS: inducible nitric oxide synthase; NF-κB: nuclear factor kappa B; MAPK: mitogen-activated protein kinase; TARC: thymus and activation-regulated chemokine; MDC: macrophage-derived chemokine; ERK: extracellular signal-regulated kinase; n.d.: not described; TMJ: temporomandibular joint; SRBC: sheep red blood cells. PGE2: prostaglandin E2; PMNs: polymorphonuclear leukocytes; MPO: myeloperoxidase; GVHD: graft-vs-host disease; BDMC: bone-marrow derived dendritic cells; THP-1: human THP-1 macrophages; RAW 264.7: mouse macrophage cell line; n.d.: not defined.

Red Macroalgal Species	Compound or Extract (Technique)	Concentration Tested	Anti-Inflammatory Assay	Outcome	Reference
*Agardhiella ramosissima*	Sulfated polyssacharide	30 mg·kg^−1^	Swiss mice: carrageenan, dextran, serotonin and histamine induced paw oedema; carrageenan induced peritonitis	Reduced neutrophil migration in peritonitis model; Reduced paw oedema	[121]
*Amansia multifida*	Ethanol:water 7:3 (SLE)	2.5, 5, 10 mg·kg^−1^	Swiss mice: carrageenan induced peritonitis; carrageenan induced paw oedema.	Reduced neutrophil migration in peritonitis model; Reduced paw oedema	[162]
	Lectin	0.1, 0.3, 1 mg·kg^−1^	Swiss mice: carrageenan-induced peritonitis; carrageenan-, compound 48/80-, histamine- and PGE2-induced paw oedema	Inhibition of paw oedema for all stimulators, inhibition of neutrophil migration, increase in GSH levels, inhibition of TNF-α and IL-1β	[163]
*Asparagopsis taxiformis*	Water (SLE)	n.d.	Enzymatic activity	COX-2 inhibition	[171]
	Ethanol:water 96:4 (SLE)	1 mg·mL^−1^	Enzymatic activity	COX-2 inhibition	[172]
*Bryothamnion triquetrum*	Lectin	1, 5, 10 mg·kg^−1^	Swiss mice: carrageenan induced peritonitis; carrageenan and dextran induced paw oedema	Reduction of oedema; Reduction of leukocyte infiltrations. IL-1β and TNF-α inhibition	[169]
*Chondrus crispus*	Lipid extract	3 μg·mL^−1^ total fatty acids	LPS-stimulated THP-1	TLR1, TLR2, TLR4, TLR8, TRAF5, TRAF6, TNFSF18, IL6R, IL23, CCR1, CCR4, CCL17, STAT3, MAP3K1 downregulation	[159]
*Chondrus verrucosus*	Polyssacharides	100, 200, 400 µg·mL^−1^	A23187-stimulated RBL-2H3 cells	Degranulation of basophils inhibition	[114]
*Coelarthrum muelleri*	MeOH (SLE)	n.d.	Carrageenan-induced rat paw oedema	Reduction of oedema	[164]
*Delesseria sanguinea*	Sulfated Polysaccharides	n.d.	Enzymatic activity	Elastase inhibition	[173]
*Dichotomaria obtusata*	MeOH (SLE)	0.0005–2 mg·ear^−1^ 12.5–100 mg·kg^−1^	Cenpalab mice: croton oil induced ear oedema	Reduced ear oedema	[174]
	Water (SLE)	12.5, 25 and 50 mg·kg^−1^	Cenpalab mice: TPA-induced ear oedema	Reduction of oedema	[175]
*Digenia simplex*	Polysaccharide	10, 30 and 60 mg·kg^−1^	Swiss mice: carrageenan-induced peritonitis; carrageenan-dextran-, serotonin-, histamine- and bradykinin-induced paw oedema	Reduction of oedema; Reduction of leukocyte infiltrations; Inhibition of IL-1β and TNF-α	[116]
*Eucheuma cottonii*	MeOH:Water 1:1 (SLE)	150, 300 mg·kg^−1^	Sprague-Dawley rats: ovalbumin induced asthma	Reduced lung inflammation and blood cells migration and positively modulated several inflammatory markers	[165]
	MeOH:Water 1:1 (SLE)	150, 300 mg·kg^−1^	Sprague-Dawley rats: SRBC induced paw oedema	Pro-inflammatory at 150 mg·kg^−1^ Anti-inflammatory at 300 mg·kg^−1^	[165]
*Eucheuma denticulatum*	Ethanol (SLE) and SPE fractions	1–100 µg·mL^−1^	TNF-γ- and LPS-stimulated RAW264.7	Non-inflammatory morphology conserved; NO, TNF-α, IL-1β, IL-6 and MCP-1 inhibition	[176]
*Gelidium amansii*	Cellulose microfibril	n.d.	HaCaT	JNK1/2 and p38 inhibition	[119]
	Cellulose nanocrystal	n.d.	UVB-stimulated HaCaT	AP-1, COX-2, c-Jun translocation inhibition; phosphorilation of ERK1/2/B-Raf, JNK1/2/MKK4/7, Akt and EGFR inhibition	[120]
	Cellulose nanocrystal	40 and 200 mg·kg^−1^	UVB-stimulated mice	Epidermal thickening and COX-2 inhibition	[120]
	Hot water (SLE) partitioned with Ethanol	n.d.	LPS-stimulated RAW264.7	Reduced TNF-α, IL-1β and IL-6	[177]
*Gelidium crinale*	Sulfated galactan	0.01, 0.1 and 1 mg·kg^−1^	Wistar rats: several stimulatory agents of paw oedema	Reduced paw oedema	[126]
*Gelidium pacificum*	Sulfated Polysaccharides	0–300 µg·mL^−1^	LPS-stimulated THP-1	NO, TLR4, MyD88 and TRAF6 inhibition	[127]
*Gelidium sesquipedale*	Ethanol (SLE)	1 mg·mL^−1^	Enzymatic activity	COX-2 inhibition	[178]
*Gloiopeltis furcata*	Ethyl acetate (SLE)	50 µg·mL^−1^	LPS-stimulated RAW264.7	NO, PGE2, IL-6, TNF-α inhibition	[161]
*Gracilaria birdiae*	Sulfated Polysaccharide	5, 10, 20 mg·kg^−1^	Wistar rats: carrageenan-induced peritonitis; carrageenan- and dextran-induced paw oedema	Reduced paw oedema and leukocyte migration	[122]
*Gracilaria caudata*	Sulfated Polysaccharides	2.5, 5 and 10 mg·kg^−1^	Swiss mice: carrageenan, dextran, bradykinin and histamine paw oedema; carrageenan induced peritonitis	Reduction of oedema (some inducers). Reduction of leukocyte infiltrations. IL-1β and TNF-α inhibition	[123]
*Gracilaria changii*	MeOH (SLE)	10 µg·mL^−1^	PMA-differentiated U937	Inhibition of TNF-α and IL-6	[179]
*Gracilaria cornea*	Sulfated Polysaccharides	3, 9, 27 mg·kg^−1^	Wistar rats: carrageenan-induced peritonitis; carrageenan and dextran induced paw oedema	Leukocyte infiltration and oedema reduction.	[124]
*Gracilaria lemaneiformis*	Agaro-oligosaccharides	12.5, 25, 50 µg·mL^−1^	LPS-stimulated RAW264.7	NO, PGE2, COX-2, TNF-α, IL-1β and IL-6 inhibition	[117]
	Agaro-oligosaccharides	12.5, 25, 50 µg·mL^−1^	LPS-stimulated zebrafish embryo	NO and ROS inhibition	[117]
*Gracilaria opuntia*	Sulfated galactan	n.d.	Enzymatic activity	Inhibition of COX-2 and 5-LOX	[129]
	2-acetoxy-2-(5-acetoxy-4-methyl-2-oxotetrahydro-2H-pyran-4-yl)ethyl 4-(3-methoxy-2-(methoxymethyl)-7-methyl-3,4,4a,7,8,8a-hexahydro-2H-chromen-4-yloxy)-5-methylheptanoate	n.d.	Enzymatic activity	Inhibition of COX-2 and 5-LOX	[144]
	3-(2-ethyl-6-((3Z,7Z)-1,2,5,6-tetrahydroazocin-5-yl)hexyl) morpholin-6-one	n.d.	Enzymatic activity	Inhibition of COX-2 and 5-LOX	[146]
	2-(3-ethyl-9-(2-methoxyethoxy)-1-oxo-2,3,4,9-tetrahydro-1H-xanthen-2-yl) ethyl-5-hydroxy-9-methoxy-7,8-dimethyl-8-(5-methylfuran-2-yl) nona-3,6-dienoate	n.d.	Enzymatic activity	Inhibition of 5-LOX	[145]
*Gracilaria salicornia*	Ethyl acetate:MeOH 1:1 (SLE)	n.d.	Enzymatic activity	Inhibition of COX-2 and 5-LOX	[143]
	Methyl-16(13–>14)-abeo-7-labdene-(12-oxo) carboxylate	n.d.	Enzymatic activity	Inhibition of COX-2 and 5-LOX	[143]
	4′-[10′-[7-hydroxy-2,8-dimethyl-6-(pentyloxy)-2H-chromen-2-yl]ethyl]-3′,4′-dimethyl-cyclohexanone	n.d.	Enzymatic activity	Inhibition of COX-2 and 5-LOX	[180]
	3′-[10′-(8-hydroxy-5-methoxy-2,6,7-trimethyl-2H-chromen-2-yl)ethyl]-3′-methyl-2′-methylene cyclohexyl butyrate	n.d.	Enzymatic activity	Inhibition of COX-2 and 5-LOX	[180]
	Salicornolides A-C	n.d.	Enzymatic activity	Inhibition of COX-2 and 5-LOX	[142]
*Gracilaria* sp.	Lipid extract	100 µg·mL^−1^	LPS-stimulated RAW264.7	NO inhibition	[181]
	Ethanol (SLE)	5 and 10% (*w*/*w*) of cream	UVB-irradiated mice	Reduction of epidermal erosion and thickening induced by UVB radiation	[182]
*Gracilaria verrucosa*	(5Z,13E)-(8R,12R,15S)-15-Hydroxy-9-oxoprosta-5,13-dienoic acid	20 µg·mL^−1^	LPS-stimulated RAW264.7	NO, TNF-α, IL-6 reduction	[139]
	Methyl-(5Z,13E)-(8R,12R,15S)-15-hydroxy-9-oxoprosta-5,13-dienoate	20 µg·mL^−1^	LPS-stimulated RAW264.7	NO, TNF-α, IL-6 reduction	[139]
	(E)-(8R,12R,15S)-15-Hydroxy-9-oxoprost-13-enoic acid	20 µg·mL^−1^	LPS-stimulated RAW264.7	NO, TNF-α, IL-6 reduction	[139]
	(Z)-(8R,12S)-9,15-Dioxoprost-5-enoic acid	20 µg·mL^−1^	LPS-stimulated RAW264.7	NO, TNF-α, IL-6 reduction	[139]
	(2R,3S)-2-Formamido-1,3-dihydroxyoctadecane	20 µg·mL^−1^	LPS-stimulated RAW264.7	IL-6 reduction	[139]
	(E)-9-Oxohexadec-10-enoic acid	20 µg·mL^−1^	LPS-stimulated RAW264.7	NO, TNF-α, IL-6 reduction	[139]
	10-Oxohexadecanoic acid	20 µg·mL^−1^	LPS-stimulated RAW264.7	NO, TNF-α, IL-6 reduction	[139]
	(E)-(S)-10-Hydroxyhexadec-8-enoic acid	20 µg·mL^−1^	LPS-stimulated RAW264.7	NO, TNF-α, IL-6 reduction	[139]
	(E)-10-Oxooctadec-8-enoic acid	20 µg·mL^−1^	LPS-stimulated RAW264.7	NO, TNF-α, IL-6 reduction	[139]
	(E)-9-Oxooctadec-10-enoic acid	20 µg·mL^−1^	LPS-stimulated RAW264.7	NO, TNF-α, IL-6 reduction	[139]
	(E)-(R)-10-Hydroxyoctadec-8-enoic acid	20 µg·mL^−1^	LPS-stimulated RAW264.7	NO, TNF-α, IL-6 reduction	[139]
	10-Oxooctadecanoic acid	20 µg·mL^−1^	LPS-stimulated RAW264.7	NO, TNF-α, IL-6 reduction	[139]
	11-Oxooctadecanoic acid	20 µg·mL^−1^	LPS-stimulated RAW264.7	NO, TNF-α, IL-6 reduction	[139]
	12-Oxooctadecanoic acid	20 µg·mL^−1^	LPS-stimulated RAW264.7	NO, TNF-α, IL-6 reduction	[139]
*Grateloupia elliptica*	Ethyl acetate (SLE)	50 µg·mL^−1^	LPS-stimulated RAW264.7	NO, PGE2, IL-6, TNF-α inhibition	[161]
*Grateloupia lanceolata*	Ethanol:water 7:3 (SLE)	0–100 µg·mL^−1^	LPS-stimulated RAW264.7	NO, IL-1Β, p38 MAPK/ERK/JNK and NF-κB inhibition	[154]
*Grateloupia turuturu*	Lipids	12.5–250 µg·mL^−1^	Enzymatic activity	COX-2 inhibition	[183]
	Hydroethanolic and water SLEs	0.02–0.2 mg·mL^−1^	LPS-stimulated RAW264.7	NO inhibition	[184]
*Hypnea cervicornis*	Agglutinin	0.3–3 mg·kg^−1^	Wistar rats: Zymosan-induced arthritis	Reduced leukocyte influx. iNOS and TNF-α inhibition.	[168]
	Agglutinin	0.1–10 mg·kg^−1^	Wistar rats: carrageenan-, ovalbumin- and PGE2-induced inflammation	Inhibition of neutrophil migration; increase in NO	[185]
*Hypnea musciformis*	Sulfated polysaccharide	10 mg·kg^−1^	Swiss mice: carrageenan-induced peritonitis; carrageenan- and dextran-induced paw oedema	Reduced leukocyte influx; Reduced paw oedema; IL-1β inhibition	[125]
*Kappaphycus alvarezii*	Sulfated galactan	n.d.	Enzymatic activity	Inhibition of COX-2 and 5-LOX	[129]
	(3S, 4R, 5S, 6Z)-3-((R)-hexan-2′-yl)-3,4,5,8-tetrahydro-4-methyl-2H-oxocin-5-yl acetate	n.d.	Enzymatic activity	Inhibition of 5-LOX	[186]
	2-ethyl-6-(4-methoxy-2-((2-oxotetrahydro-2H-pyran-4-yl)methyl)butoxy)-6-oxohexyl 5-ethyloct-4-enoate	n.d.	Enzymatic activity	Inhibition of 5-LOX	[187]
	4-(2-chloroethyl)-5-7-(methoxymethyl) undec-3-enyl) cyclooct-4-enone	n.d.	Enzymatic activity	Inhibition of COX-2 and 5-LOX	[188]
*Laurencia glandulifera*	Neorogioltriol	0.5–1 mg·kg^−1^	Rats: carrageenan induced paw oedema	Reduction of paw oedema	[141]
	Neorogioltriol	12.5–62.5 µM	LPS-stimulated RAW264.7	NO, COX-2, TNF-α and NF- kB inhibition	[141]
*Laurencia okamurae*	Ethyl acetate (SLE)	25 µg·mL^−1^	LPS-stimulated RAW264.7	NO, PGE2, IL-6, TNF-α inhibition	[161]
*Laurencia snackeyi*	5β-hydroxypalisadin B	0.25, 0.1 and 1 µg·mL^−1^	LPS-induced zebrafish embryo	NO and ROS inhibition; Improved survival, heart rate and yolk sac oedema size	[189]
*Lithothamnion muelleri*	Whole seaweed	1% (*w*/*w*) in diet	GVHD mice model	Reduced IFN-γ, TNF-α, CCL2, CCL3, CCL5.	[190]
*Melanothamnus afaqhusainii*	MeOH (SLE)	n.d.	Carrageenan- induced rat paw oedema	Reduction of oedema	[164]
*Palmaria palmata*	Ethyl acetate LLE of MeOH:Chloroform (SLE)	n.d.	LPS-stimulated RAW264.7	NO and iNOS inhibition	[191]
	Thermolysin-digested water extract	100–1000 µg·mL^−1^	LPS-stimulated RAW264.7 and carrageenan-induced paw oedema	Reduction of NO, TNF-α and IL-6; Reduction of paw oedema	[157]
	Phenolic Extract (LLE of MeOH SLE)	25, 50 and 100 µg·mL^−1^	LPS-stimulated primary human neutrophils	Reduction of ROS, NO, MPO, IL-8, IL-1β, IL-6 and TNF-α; Downregulation of TLR4	[158]
	Lipid extract	3 μg·mL^−1^ total fatty acids	LPS-stimulated THP-1	TLR1, TLR2, TLR4, TLR8, TRAF5, TRAF6, TNFSF18, IL6R, IL23, CCR1, CCR4, CCL17, STAT3, MAP3K1 downregulation; IL-6 and IL-8 inhibition	[159]
*Polysiphonia morrowii*	Bis (3-bromo-4,5-dihydroxybenzyl) ether	0.1, 1, 2 µM	LPS-stimulated RAW264.7	NO, iNOS, COX-2, PGE2, TNF-α, IL-6 and IL-1β inhibition	[192]
	3-bromo-5-(ethoxymethyl)-1,2-benzenediol	12.5–50 µM	LPS-stimulated RAW264.7 and Zebrafish embryos	NO, ROS, iNOS, COX-2 and NF-κB inhibition	[155]
*Porphyra columbina*	Protein fraction	n.d.	Several cell lines	IL-10 elicitation; pro-inflammatory cytokines inhibition	[193]
*Porphyra dentata*	MeOH (SLE)	25, 50, 100, 200 µg·mL^−1^	LPS-stimulated RAW264.7	NO reduction	[150]
	MeOH (SLE)	50, 100, 200 µg·mL^−1^	LPS-stimulated RAW264.7	iNOS inhibition	[150]
	MeOH (SLE)	200 µg·mL^−1^	LPS-stimulated RAW264.7	NF-κB inhibition	[150]
	Catechol	6 µg·mLl^−1^	LPS-stimulated RAW264.7	NO reduction	[150]
	Catechol	1–11 µg·mL^−1^	LPS-stimulated RAW264.7	iNOS inhibition	[150]
	Catechol	11 µg·mL^−1^	LPS-stimulated RAW264.7	NF-κB inhibition	[150]
	Rutin	250 µg·mL^−1^	LPS-stimulated RAW264.7	NO reduction	[150]
	Rutin	80–250 µg·mL^−1^	LPS-stimulated RAW264.7	iNOS inhibition	[150]
	Rutin	250 µg·mL^−1^	LPS-stimulated RAW264.7	NF-κB inhibition	[150]
	Hesperidin	250 µg·mL^−1^	LPS-stimulated RAW264.7	NO reduction	[150]
*Porphyra dioica*	Lipid extract	3 μg·mL^−1^ total fatty acids	LPS-stimulated THP-1	TLR1, TLR2, TLR4, TLR8, TRAF5, TRAF6, TNFSF18, IL6R, IL23, CCR1, CCR4, CCL17, STAT3, MAP3K1 downregulation	[159]
*Porphyra* sp.	Shinorine	12.5–200 µg·mL^−1^	LPS-stimulated THP-1 and THP-1-Blue	NF-κB increase and IDO-1 suppression	[151]
	Porphyra-334	12.5–200 µg·mL^−1^	LPS-stimulated THP-1 and THP-1-Blue	NF-κB inhibition and IDO-1 suppression	[151]
*Porphyra tenera*	Several enzymatic extracts	62.5, 125 and 250 μg·mL^−1^	LPS-stimulated RAW264.7	NO reduction	[194]
*Porphyra umbilicalis*	Hydroethanolic and water SLEs	0.005–0.02 mg·mL^−1^	LPS-stimulated RAW264.7	NO reduction	[184]
*Porphyra vietnamensis*	MeOH:Water 4:1 (Soxhlet)	200 mg·kg^−1^	Wistar rats: Carrageenan-induced paw oedema	Reduction of paw oedema	[115]
	Precipitated polysaccharide	250 mg·kg^−1^	Wistar rats: Carrageenan-induced paw oedema	Reduction of paw oedema	[115]
*Porphyra yezoensis*	Aqueous protein extract (SLE)	25, 50, 100 µg·mL^−1^	HK2	MAPK and NF-κB downregulation	[152]
	MAAs (EtOH, SLE)	5, 10, 20 µg·mL^−1^	Male ICR mice (skin)	IL-1β, IL-6, IL-10, NF-κB expression reduction	[153]
*Porphyridium cruentum*	Sulfoglycolipid fraction	n.d.	Activated peritoneal mono nuclear cells from Wistar rats	Inhibition of Superoxide generation	[195]
*Porphyridium* sp.	Polysaccharide	50–500 µg·mL^−1^	HCAEC induced with angiotensin II	Inhibition of adhesion molecules and NF-κB expression; increase in aantioxidant system activity	[118]
	Polysaccharide	0.005–1% *w*/*v*	fMLP-stimulted PMNs	Inhibition of PMN chemotaxis	[196]
*Pterocladiella capillacea*	Ethanol (SLE)	1 mg·mL^−1^	Enzymatic activity	COX-2 inhibition	[178]
	Lectin	8.1 mg·kg^−1^	Wistar rats: carrageenan-induced paw oedema and peritonitis	Reduced paw oedema and leukocyte migration.	[170]
*Pyropia yezoensis*	MeOH (SLE)	40, 200, 1000 µg·mL^−1^	HaCaT induced with IFN-ϒ	TARC and MDC expression inhibition	[156]
	MeOH (SLE)	40, 200, 1000 µg·mL^−1^	HaCaT induced with TNF-α	TARC and MDC expression inhibition	[156]
	MeOH (SLE)	40, 200, 1000 µg·mL^−1^	HaCaT induced with IFN-ϒ	ERK, JNK, p38 inhibition	[156]
	MeOH (SLE)	40 µg·mL^−1^	HaCaT induced with TNF-α	ERK inhibition	[156]
	MeOH (SLE)	1000 µg·mL^−1^	HaCaT induced with TNF-α	JNK and p38 inhibition	[156]
	MeOH (SLE)	40, 200, 1000 µg·mL^−1^	HaCaT induced with IFN-ϒ	NF-κB and IkB-α inhibition	[156]
	Peptide PPY1	250–1000 ng·mL^−1^	LPS-stimulated RAW264.7	NO, ROS, iNOS, COX-2, IL-1β and TNF-α inhibition; p38 and MAPK downregulation	[160]
	Porphyran	0–100 µg·mL^−1^	C57BL/6 mice derived, LPS-stimulated BDMCs	Supression of CCR7, IL-6, IL-12 and TNF-α expression	[134]
	Porphyran	0–100 mg·kg^−1^	LPS-stimulated C57BL/6 mice	Supression of Th1 and Tc1 cells differentiation	[134]
*Sarcodia ceylanica*	Ethyl acetate LLE of Ethanol (95% *v*/*v*) SLE	10, 20, 50 µg·mL^−1^	LPS-stimulated RAW264.7	iNOS and COX-2 inhibition	[166]
	Ethyl acetate LLE of Ethanol (95% *v*/*v*) SLE	20, 50 mg·kg^−1^	Wistar rats: carrageenan-induced paw oedema	Reduced paw oedema	[166]
*Solieria filiformis*	Lectin	1, 3, 9 mg·kg^−1^	Wistar rats: carrageenan induced peritonitis; carrageenan, dextran, bradykinin, histamin and serotonin induced paw oedema.	Reduced neutrophil migration in peritonitis model; Reduced paw oedema	[167]
	Lectin	10 µg·mL^−1^	BALB/c mice splenocytes	IL-6 and IL-10 production (Th2 immune-response stimulators)	[197]
	Sulfated polysaccharide	0.03, 0.3, 3 mg·kg^−1^	Wistar rats: formalin induced TMJ inflammation	Reduced IL-1β and TNF-α	[128]
*Solieria robusta*	MeOH (SLE)	n.d.	Carrageenan- induced rat paw oedema	Reduction of oedema	[164]
*Tichocarpus crinitus*	Kappa/beta Carrageenan	n.d.	Human blood cells	Increase in IL-10	[137]
*Vidalia obtusaloba*	Vidalols A and B	n.d.	PMA-induced mouse ear oedema; enzymatic activity	Reduction of oedema and inhibition of Phospholipase A_2_	[198]

### 4.3. Extracts and Compounds from Red Macroalgae Targeting Other Mechanisms of AV

The literature search on anti-sebum compounds or extracts from Rhodophyta, even when it comes to macroalgae in general, revealed a complete absence of published studies. Despite this fact, SEPPIC (La Garenne-Colombes, France), a cosmetic company, own red seaweed-incorporating products in the market claiming to possess anti-sebum properties. The incorporation of macroalgal extracts in the skincare line product WESOURCE (SEPPIC) revealed an anti-sebum effect (34% reduction after 56 days), observed when oily skinned volunteers were tested using CONTACTICEL™ (SEPPIC, La Garenne-Colombes, France) [199], a skin-care product that contained extracts from *Acrochaetium moniliforme*, a red seaweed product under the patent WO2016162648A1. Furthermore, similar results were obtained with a brown seaweed, revealing in vivo sebum regulation—29.3% lower sebum in 23 days—providing visible mattifying effects on volunteers subjected to *Laminaria saccharina* extract [199]. Because both cosmetic products are effective in the reduction of facial sebum, using active ingredients obtained from two macroalgae, despite no scientific publications being found, at least some red and brown seaweeds may be considered to have anti-sebum properties in vivo.

Hyperlipidaemia, a condition characterized by high levels of lipids in the blood, e.g., cholesterol, was associated with patients exhibiting acne. In accordance with this, high levels of triglycerides (TG), low-density lipoprotein cholesterol (LDL), and high-density lipoprotein cholesterol (HDL) were found in the blood of both male and female subjects [200,201]. A study by Liu et al. (2017) showed the improvement of carbohydrate and lipid metabolism in rats which were hyperlipidaemia-induced by high fructose (HF) intake, when fed with a *Gelidium amansii*-supplemented diet. *Gelidium amansii* supplementation resulted in the decrease of glucose, leptin, insulin, and TNF-α blood levels [202]. Moreover, it also reduced the accumulation of hepatic lipids, namely TG and the total cholesterol (TC) content, while increasing the excretion of bile acid and faecal lipids [202]. Further studies are required to prove Rhodophyta’s anti-hyperlipidaemia properties, but evidence already shows the potential of using this biomass to reduce the lipid levels in acne patients. A significant finding in this study, besides the clear improvement in lipid metabolism, lies in the fact that TNF-α rat blood levels decreased due to *G. amansii* supplementation, corroborating previous results in which algae extract reduced TNF-α, IL-1 and IL-6 levels in LPS-stimulated RAW264.7 [177]. Because TNF-α is known to have an important role in oxidative stress, and in the initiation of inflammatory and immunological processes [203,204] which are also involved in acne pathophysiology, *G. amansii* represents an opportunity to address multi targets of AV disease.

In order to the present the knowledge and data search criteria used, no scientific papers were found concerning red seaweed-derived compounds targeting acne-involved hormones, keratinization/cornification, or comedogenesis.

The anti-sebum property of red seaweeds is a relevant topic on the AV battle, because the disease is not observed in its absence. Being a precursor of the AV cascade, it is of utmost concern to study the potential of Rhodophyta, and also other macroalgae, to inhibit the hyperproduction of sebum, and how it may contribute to the avoidance of the stimulation of sebaceous glands.

## 5. Conclusions

Compounds found in macroalgal species are valuable for pharmaceuticals, cosmetics, feeds, and foods due to their valuable and recognized bioactive properties. Red macroalgal species are suitable candidates to the field of drug discovery, because they are abundant in fatty acids, pigments, phenolic compounds, and polysaccharides with therapeutic interest.

A literature revision on antibacterials that inhibit the growth of *Cutibacterium acnes* and *Staphylococcus epidermidis* found in the phylum Rhodophyta revealed a limited number of research papers, and more importantly, that a very large portion of Rhodophyta species remains unstudied for this purpose. Despite the relatively few reports, the available research showed that red seaweed possesses potent anti-*C. acnes* and *S. epidermidis* activity. On the contrary, the given number of studies concerning the anti-inflammatory capacity of isolated compounds and extracts, both in vitro and in vivo, revealed a significant potential for those macroalgae to be used in the mitigation of inflammatory pathways in AV. Unfortunately, there is as yet no published data concerning research on the other three AV pathophysiological targets (hormones, keratinization, or sebum), but some cosmetic anti-sebum products produced with macroalgae extracts are already in the market, confirming that at least some Rhodophyta compounds have anti-sebum properties, which should trigger further research into this topic. Red seaweed-derived compounds are the most studied in anti-inflammatory contexts, and on the contrary, there is as yet no knowledge related to AV-related hormones and comedogenesis. As we mentioned, and because AV pathophysiology is multi-factorial, there is a need to research the diversity of red algae compounds with antibacterial activity in acne-related bacteria, and to perform in vivo clinical trials of anti-inflammatory compounds in AV patients, in vitro assays to understand inhibition of hyperplasia by the compounds, and hormonal and comedogenesis evaluations to better understand the mechanisms of action.

Red seaweed derived compounds, namely polysaccharides, terpenoids, fatty acids, and phenolic compounds, represent good sources of antibacterial and anti-inflammatory compounds with the potential to be applied in acne vulgaris research for new treatments. In a pathology with great psychological and high-cost impacts in the society, those compounds represent the possibility to alleviate the consequences of the most prevalent skin disease.
